# Sexual dimorphism and reproductive biology of the Asian bockadam snake (*Cerberus schneiderii*) in West Java

**DOI:** 10.1038/s41598-022-25007-6

**Published:** 2022-12-01

**Authors:** Alamsyah E. N. Herlambang, Mirza D. Kusrini, Amir Hamidy, Evy Arida, Mumpuni, Awal Riyanto, Richard Shine, Daniel Natusch

**Affiliations:** 1 Laboratory of Herpetology, Museum Zoologicum Bogoriense, Research Center for Biosystematics and Evolution, Organization Research of Life Sciences and Environment, Research and Innovation Agency of Indonesia, Gd. Widyasatwaloka, Cibinong Science Center, Jl. Raya Jakarta, Bogor, Indonesia; 2 Faculty of Forestry and Environment, IPB University, Dramaga, Bogor, Indonesia; 3 Research Center for Applied Zoology, Organization Research of Life Sciences and Environment, Research and Innovation Agency of Indonesia, Gd. Widyasatwaloka, Cibinong Science Center, Jl. Raya Jakarta, Bogor, Indonesia; 4 School of Natural Sciences, Macquarie University, Sydney, NSW 2109 Australia

**Keywords:** Ecology, Zoology, Environmental sciences

## Abstract

Although they are among the most abundant snakes on Earth, and are heavily exploited for their skins and meat, Asian bockadams (or “dog-faced water snakes”, *Cerberus schneiderii*) have attracted relatively little study across their wide geographic range. Based on dissection of 3,382 snakes brought to processing facilities in and around the city of Cirebon in West Java, Indonesia, we document facets of the biology of these mangrove-dwelling aquatic homalopsids. Females attain larger body sizes than do males, and are heavier-bodied (due in part to greater fat reserves) but have shorter tails relative to snout-vent length. Males showed testicular enlargement late in the year (August-November) but both reproductive and non-reproductive females were found year-round. Litters were large (3 to 45 offspring), especially in larger females. The commercial harvest falls mainly on adult snakes of both sexes, with seasonal variation in sex ratios. Life-history traits such as early maturation and frequent production of large litters render this species resilient to commercial harvesting. Future research should explore reasons for strong variation among facilities in the sex ratios of snakes, potentially identifying ways to focus the harvest on the sex (males) whose numbers are less critical for population viability.

## Introduction

A strong focus of ecological research on temperate-zone systems, and on terrestrial rather than aquatic taxa, has resulted in a situation whereby even highly abundant and heavily-utilised tropical aquatic species have attracted relatively little attention from scientists^1^. One spectacular example involves mangrove-dwelling snakes of the family Homalopsidae (formerly regarded as a subfamily within the larger entity Colubridae^2^). In a paper showing that snakes previously allocated to “*Cerberus rynchops*” comprised four species-level taxa (one of which is *C. schneiderii,* the focus of the current paper), Murphy et al.^3^ suggested that snakes of this lineage may be among the most abundant aquatic ophidian species on the planet. Additionally, these snakes are heavily harvested for skin, meat, and medicinal value over much of their extensive geographic range^4–6^. Notwithstanding this abundance and commercial importance, available information on ecological traits of *Cerberus schneiderii* is derived from studies in only a few locations, and published reports are contradictory about even fundamental biological attributes such as sexual dimorphism and seasonality of reproduction (reviewed by Murphy^6^).

The dearth of research on tropical homalopsids reflects the mangrove habitats in which they live. Even in places where they are abundant, the snakes spend most of their time in muddy banks and dense vegetation and hence are rarely seen by observers^6,7^. Mark-recapture and radiotelemetry methods can overcome such problems but require substantial investment of time and effort^8,9^. Another approach is to sample specimens that are collected and killed for commercial use^10–12^. This technique can provide large datasets in a short amount of time, does not increase collecting pressure or stress on wild populations, and has the additional benefit of clarifying whether or not the commercial harvest of snakes is sustainable. Such inferences can come both from demographic traits of the offtake (e.g., to what degree is the harvest based on adults vs. juveniles, and males vs. females?), reproductive rates of the population (is recruitment rate high enough to offset culling?), and longterm trends in size structure (is harvesting driving down mean ages and thus, body sizes?)^11,12^. Additionally, the large sample sizes obtainable from commercial processing facilities enable accurate documentation of underlying biological traits of the species involved. For example, pioneering studies on reticulated pythons in Sumatra provided the first extensive information on issues such as reproductive seasonality and the influence of sex and body size on diets and reproductive output in these giant snakes^13–15^.

In the present study, we examined > 3000 specimens of *Cerburus schneiderii* that were collected and killed for their skins, meat, and medicinal products in five processing facilities in and near the city of Cirebon in West Java, Indonesia. The present analysis is part of a larger study of snakes harvested in this region.

## Materials and methods

### Study species

**Figure 1:**
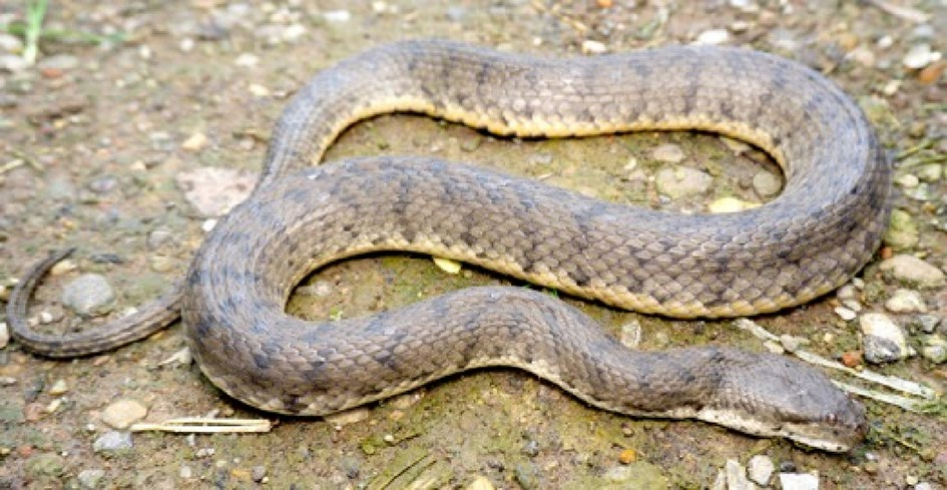
The Asian bockadam, *Cerberus schneiderii*.

Like most homalopsids, *C. schneiderii* is a heavy-bodied large-headed aquatic snake commonly encountered in mangrove ecosystems^6^ (Fig. 1). Many authors have commented on the species’ abundance, nocturnal habits, and preference for muddy habitats^16,17^. These snakes have been recorded to eat a wide variety of fishes, especially gobioids, primarily in shallow water (reviewed by Murphy^6^). The only detailed ecological studies of *C. schneiderii* comprise mark-recapture works on a Malaysian population^8^ and a Singapore population^9^. Both of these studies reported sedentary habits, rapid growth, high abundance and aseasonal reproduction. In contrast, other studies have inferred seasonal schedules of reproduction: parturition is thought to occur in May to July in Myanmar^18^, March to June in the Philippines^19^ and November to February on Komodo Island^20^. Size at birth is around 116–160 mm snout-vent length (SVL)^6,20^ and sexual maturity in females has been reported to range from around 550–570 mm SVL, followed by the production of litters containing 3–45 offspring (reviewed by Murphy^6^). Maximum body sizes appear to vary among populations, from around 750 mm in Komodo^20^ and 900 mm in the Philippines^21^ to > 1 m in Java and Myanmar^6^.

### Study area

Cirebon (6° 44ʹ N, 108° 33ʹ E) is a port city on the western coast of the island of Java, in Indonesia, approximately 300 km from the capital Jakarta. The city houses around 2.5 million people. We gathered data at six processing facilities, all of which obtain snakes from local people. We obtained data from all months of the year, with sample sizes > 200 in every month and no clear seasonal peak in numbers (e.g., the two highest counts were 528 in February and 410 in July). Of the total sample, 2,591 snakes (77%) were obtained in 2021, with fewer in 2020 (455 = 13%) and 2019 (336 = 10%). Snakes and lizards of several species are killed to obtain products such as meat, skins and natural remedies^11^. Most of the *C. schneiderii* that we examined (87%) came from a single facility (Mukti).

### Methods for collection of data

After snakes were humanely killed by staff at the processing facilities, we measured (snout-vent length [SVL] and tail length) and weighed the animals. The skin was then removed by staff, and we examined the carcass to score sex (based on gonads), fat-body mass (based on a subjective 4-point scale from 0 to 3), and reproductive condition. Females were scored as juvenile if they had thin straplike oviducts, and no ovarian follicles greater than 10 mm in length. Males were scored as juvenile if the efferent ducts were clear, and as adult if the efferent ducts were opaque and convoluted due to the presence of sperm^11,12^. We recorded numbers and sizes of ovarian follicles and oviductal embryos in females, and the length and width of the right testis in males and vitellogenic follicles in females (see^11,12^ for details). We then returned the carcass to the processing-facility staff.

### Methods for analysis of data

Using the program JMP Pro 16.0 (SAS Institute, Cary, NC), we compared sex ratios to a null hypothesis of 50:50 using contingency-table analysis. Variation in sex ratios of snakes among processing facilities, and among months, was examined using nominal logistic regression. For continuous variables, we ln-transformed data on SVL, body mass and gonadal volumes to improve normality and variance homogeneity. We examined sex differences in mean body size and in fat-body scores using ANOVA with sex as the factor, and processing facility as a random factor (to reduce pseudoreplication). Sexual dimorphism in body shape (mass and tail length relative to SVL) was assessed with ANCOVA, using sex and ln SVL as factors (plus their interaction), processing facility as a random variable, and ln mass or tail length as dependent variables.

We estimated the volume of testes and ovarian follicles from their length and width based on the formula for a prolate spheroid (4/3*π (half testis length) * (half testis width)^2^^22^. To examine seasonality, we used month as the factor in nominal logistic regressions (for reproductive state in females) or ANOVAs (for volumes of testes and follicles). Processing facility was included as a random factor in the latter analysis. The relationship between maternal body size and fecundity was described with linear regression analysis, and the relationship between maternal body size and reproductive state was assessed using nominal logistic regression.

### Ethics declaration

The research was carried out under BRIN issued permits: B2252/IPH.1/KP.06.01/VI/2019, B4033/IPH.1/KP.06.01/X/2019, B2342/IPH.1/KP.06.01/VI/2019, B502/IPH.1/KP.06.01/II/2020, and B-51/IPH.1/KP.06.01/I/2020. We gathered data from an existing trade; no live animals were used for the purpose of this research (i.e., research was undertaken on animals already killed for trade). No humans were part of this study. All work was conducted in accordance with ARRIVE guidelines.

## Results

### Sex ratio

**Figure 2:**
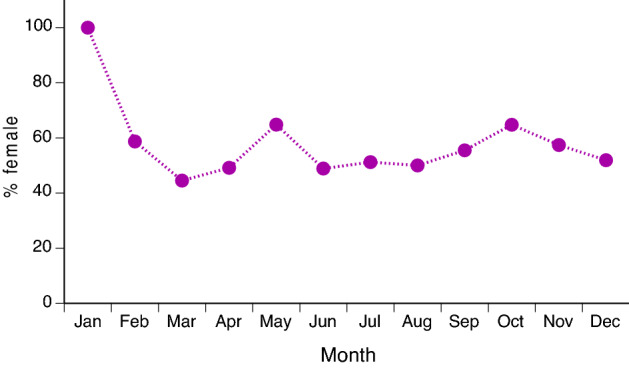
Sex ratio (proportion of snakes that were female) in samples of the Asian bockadam (*Cerberus schneiderii*) examined at processing facilities near the city of Cirebon in West Java.

The overall sex ratio was female-biased (56%; versus a null of 50:50, χ^2^ = 60.95, 1 df, *P* < 0.0001). Females outnumbered males strongly at Lilis (100% female), Tosin (95% female) and Daru (93% female) and showed a male bias at Tasrip (40% female) but exhibited a more equal sex ratio at the other sites including Mukti, the site from which > 85% of snakes were obtained (54% female). This spatial variation in sex ratio was statistically significant (logistic regression χ^2^ = 246.70, 5 df, *P* < 0.0001). Sex ratio also varied among months (χ^2^ = 258.34, 11 df, *P* < 0.0001) due to more highly female-biased samples in January in contrast to equal or male-biased sex ratios in the rest of the year (Fig. 2). Most snakes that we examined were adult (1561 of 1918 females, = 81%; 1356 of 1464 males, = 93%), especially in males (sex effect on % adult: χ^2^ = 106.75, 1 df, *P* < 0.0001).

### Sexual dimorphism

**Figure 3:**
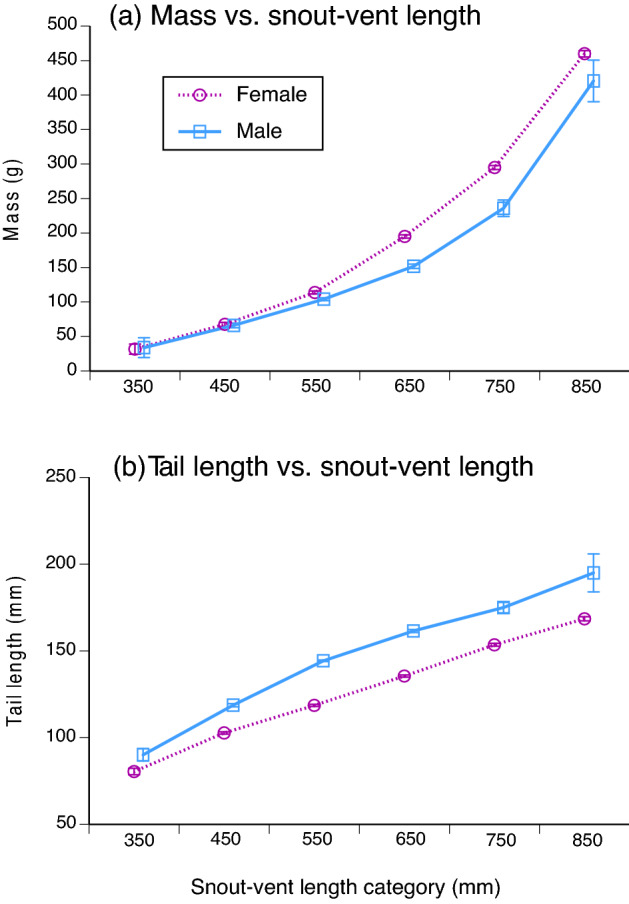
Sexual dimorphism in samples of the Asian bockadam (*Cerberus schneiderii*) examined at processing facilities near the city of Cirebon in West Java. The upper panel (**a**) shows sex-based divergence in body mass relative to snout-vent length (SVL), whereas the lower panel (**b**) shows divergence in tail length relative to snout-vent length. Points show mean values and associated standard errors for each 100-mm interval in snout-vent length, to allow clear visualisation of trends, but statistical tests in the text use continuous data rather than SVL intervals.

Females averaged larger than males in snout-vent length (adult snakes only, mean SVLs of 63.3 vs. 56.3 cm; ln SVL *F*_1,2900_ = 356.22, *P* < 0.0001; ln mass *F*_1,2905_ = 621.75, *P* < 0.0001) and were heavier-bodied than were males at the same snout-vent length (ANCOVA interaction sex*SVL on ln-transformed data, *F*_1,3360_ = 54.93, *P* < 0.0001; see Fig. 3a). Part of this disparity between the sexes was due to heavier fat-bodies in females (treating fat-body scores as a continuous variable, ANOVA *F*_1,3332_ = 96.30, *P* < 0.0001). Relative to SVL, males had longer tails than did females (ANCOVA *F*_1,1970_ = 123.35, *P* < 0.0001; see Fig. 3b).

### Seasonality of reproduction

**Figure 4:**
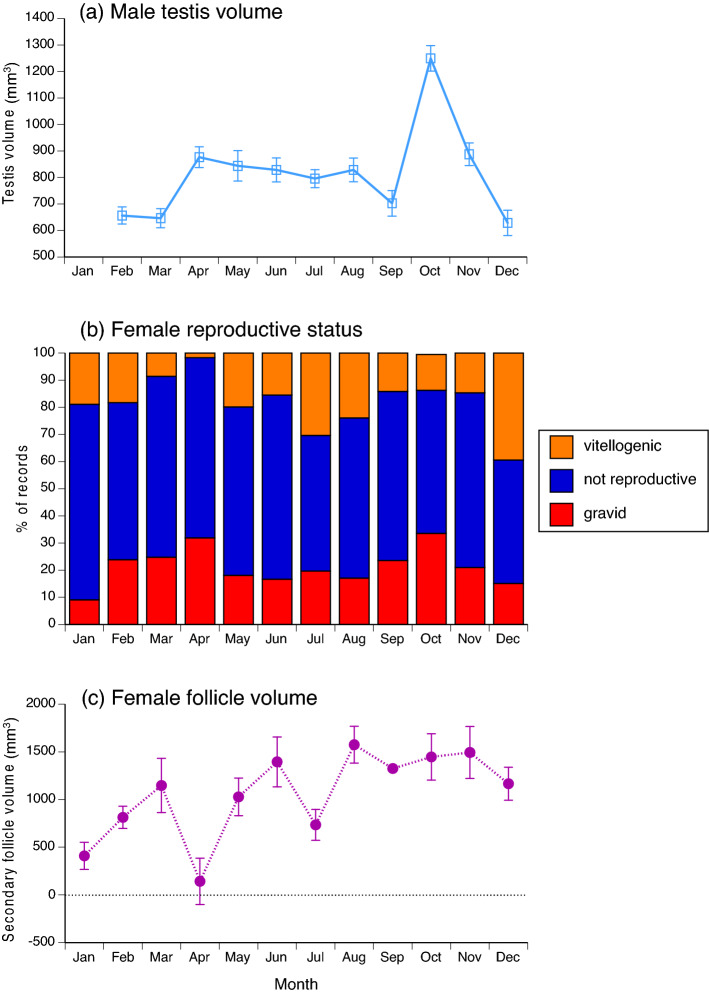
Monthly variation in reproductive traits of Asian bockadams (*Cerberus schneiderii*) examined at processing facilities near the city of Cirebon in West Java. The upper panel (**a**) shows ln-transformed values for volume of the right testis in males. The middle panel (**b**) shows relative numbers of adult female snakes that were classed as vitellogenic, gravid, or non-reproductive. The lower panel (**c**) shows ln-transformed values for mean volume of enlarged ovarian follicles. Points in (**a**) and (**c**) show mean values and associated standard errors for each month.

**Figure 5:**
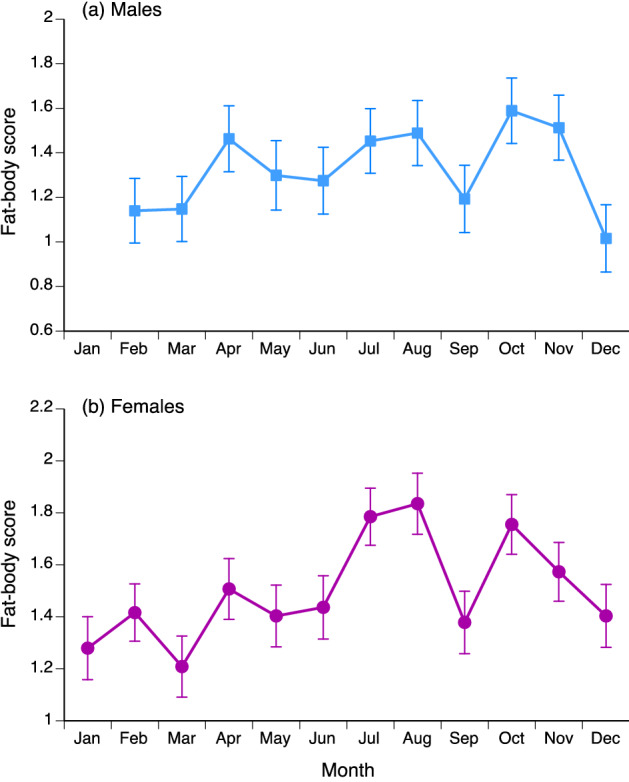
Monthly variation in size of the fat bodies in Asian bockadams (*Cerberus schneiderii*) examined at processing facilities near the city of Cirebon in West Java. The upper panel (**a**) shows values for males, and the lower panel (**b**) shows values for females. Points show mean values and associated standard errors for each month. Note that fat-body size was scored on a 4-point scale (0–3) but is treated as a continuous variable here to show patterns.

Gonad condition and dimensions varied seasonally in both sexes. In males, the significant variation among months in volume of the right testis in adult male snakes (*F*_10,1338_ = 12.09, *P* < 0.0001) reflected an increase in testis size from September to October, followed by a substantial decrease in size (Fig. 4a). Although females also exhibited significant monthly variation in follicle sizes (*F*_11,236.9_ = 8.78, *P* < 0.0001) and reproductive state (nominal logistic regression, χ^2^ = 118.16, 33 df, *P* < 0.0001), seasonal patterns were less clear. All three categories of reproductive state (non-reproductive, vitellogenic, gravid) were present year-round (Fig. 4b) with little overt pattern. The small mean size of follicles in April (Fig. 4c) is based on a sample size of 15 vitellogenic animals and thus is unlikely to be an artefact of insufficient sampling. Reproduction in females may be aseasonal, or may exhibit multiple peaks. Mass of the fat-bodies (as ranked by our scoring system) varied strongly among months in males (*F*_10,1372_ = 12.90, *P* < 0.0001), and was higher in the period when testes were enlarged (October: Fig. 5a). Monthly variation in fat-body scores was also significant in females (*F*_11,1512_ = 9.86, *P* < 0.0001) with a peak mid-year (Fig. 5b).

### Body size at maturity

Snout-vent lengths of juvenile snakes ranged from 290 to 550 mm (females) and 324 to 490 mm (males), whereas adult snakes were 420 to 960 mm SVL (females) and 400 to 890 mm SVL (males).

### Fecundity

**Figure 6:**
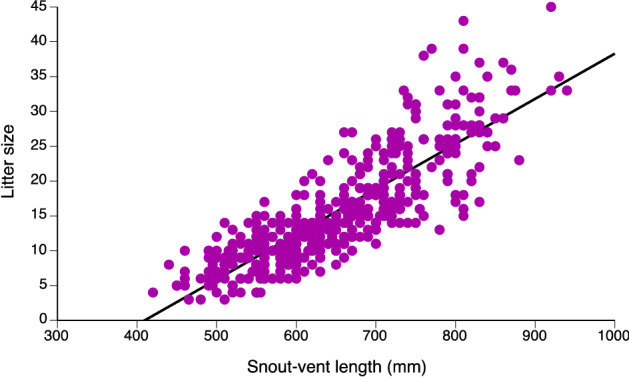
The relationship between maternal body size (snout-vent length) and fecundity in Asian bockadams (*Cerberus schneiderii*) examined at processing facilities near the city of Cirebon in West Java.

Litter sizes based on oviductal embryos ranged from 3 to 45 (mean = 14.56, SE = 0.42; median litter = 13; Fig. 6) and increased with maternal body length (*r*^2^ = 0.51, *N* = 351, *P* < 0.0001; Fig. 6). Around 40% of adult females were reproductive (18% vitellogenic, 22% gravid) at the time of collection, regardless of season (Fig. 4b).

### Reproductive frequency

Among females that we classed as adults, the proportion of reproductive animals increased with maternal body length (nominal logistic regression, χ^2^ = 55.64, 8 df, *P* < 0.0001), comprising 26% of animals < 500 mm SVL but rising to 61% of animals > 800 mm SVL. The proportion of animals that were reproductive at the time they were collected was > 45% among all animals > 600 mm SVL, but lack of a clear reproductive season prevents us from estimating the proportion of females that reproduce each year.

## Discussion

Asian bockadams, *Cerberus schneiderii*, are abundant in anthropogenically disturbed habitats of West Java, and are killed in large numbers for their meat, skins, and medicinal value. Our dissections confirm that both sexes are taken for this purpose, but that females are larger than males and are taken (or retained for sale) more frequently (perhaps because of the higher commercial value of larger snakes). Body sizes at maturation are small and are attained at an early age, based on mark-recapture studies of growth rate^8,9^. Litters are large, and produced year-round. These traits render *C. schneiderii* resilient to commercial harvesting.

Published reports of sexual dimorphism and reproductive seasonality in *C. schneiderii* are contradictory, likely reflecting both geographic variation and insufficient sampling. First, we explore sampling issues. The substantial variation in sex ratios and body sizes of our snakes among months, and among the six processing facilities, suggests that such traits can only be robustly sampled by examining specimens from multiple locations over an extended period. Any small sample taken from a single site or over a short period of time may fail to capture the underlying mean value for traits within a metapopulation. In keeping with that cautionary note, three studies based on large sample sizes (^8,9^ and present study) all concluded that females of this species reproduce throughout the year. In contrast, studies based on smaller numbers of specimens and/or briefer periods of sampling have inferred seasonal reproductive schedules^23^. Even with a large sample (> 3,000 snakes), our data do not allow us to definitively describe reproductive seasonality. We found clear seasonal cycles in testis volumes but not in ovarian follicle volumes nor in the monthly incidence of vitellogenic and gravid females. Future work could look in more detail at the stages of embryonic development seen in different months; the most likely scenario is that females reproduce year-round, but with a higher proportion of females are gravid in some months than in others.

To clarify the roles of underlying geographic variation versus sampling biases, studies similar to our own could be conducted in other locations where these snakes are processed in large numbers at commercial facilities. Ideally, that work should involve direct observation of snake-collecting and an analysis of economic incentives, as well as examination of carcasses at processing facilities^24^, because biases can intrude at any stage of the pathway leading from collection to processing. Although all of the facilities that we used were in the same area, the snakes they killed and processed differed in significant ways—partly reflecting purchasing policies of the traders involved.

Such biases are amplified by any spatial heterogeneity in snake populations. Research on aquatic snakes from diverse phylogenetic lineages (acrochordids, laticaudine and hydrophiine elapids, natricids, viperids) has reported that larger specimens are found in deeper water, and consume larger prey and often, different types of prey^25–30^. Although we have no detailed data to test the inference of sex-based niche divergence in *C. schneiderii*, females have wider and longer heads than do conspecific males of the same body length (Shine^28^, as “*C. rhynchops*”; and^9^). In previous studies, such head-size dimorphism has been associated with sex-based differences in the sizes and types of prey consumed^25–30^. Also, a survey of snakes collected by professional hunters in West Java found differences in mean body sizes of homalopsids as a function of waterbody type: fishponds contained larger individuals than did canals^24^. If snakes of different sexes and body sizes utilise different habitats, any sample from a single site likely may fail to capture average values for the wider population.

Nonetheless, at least some of the divergent results from previous studies may reflect genuine geographic variation. Although spatial heterogeneity in sex ratios and body sizes may affect estimates of traits such as sexual dimorphism in mean adult body sizes, that kind of bias should not affect estimates of allometric relationships in the two sexes. For example, any trend for females to have shorter tails than males, and to be more heavy-bodied (as in our own results) should be resilient to sampling regimes. Surprisingly, however, detailed study of a Singapore population of *C. schneiderii* revealed minimal sexual size dimorphism (males averaged larger, but the biggest individuals were females) and a heavier build in males than in females (opposite to our results)^9^. Likewise, Murphy’s^6^ compilation of morphological data (his Table 2, p. 77) showed substantial sexual dimorphism in relative tail length in most areas within the species’ range, but no dimorphism in Malaysian populations. That divergence must represent geographic variation rather than sampling issues. The same appears to be true for life-history traits such as size at birth^20^. These snakes thus have considerable potential for studies on interspecific variation in topics (such as mating systems) that likely influence sex differences in body shape and tail length^32,33^. Asian bockadams are abundant and occur over a wide range of climatic conditions, and they may well exhibit different mating systems and reproductive traits across that range.

The commercial harvest of *C. schneiderii* in West Java has been in place for decades, and the continuing abundance of the species testifies both to its ecological flexibility (ability to thrive in a highly modified landscape) and its resilience to that harvest. Our data show that these snakes mature at a small body size, and reproduce year-round with large litters. Hence, populations have a high intrinsic rate of increase, and are unlikely to be imperilled by an additional source of mortality (*i.e.,* the commercial harvest). If managers nonetheless wish to buffer the impacts of commercial harvesting on bockadam populations, the strong spatiotemporal variation in snake sexes and body sizes provides an obvious opportunity. Restricting harvests to some sites and/or some times of year could shift the impact of harvesting away from critical cohorts such as adult females.

We doubt, however, that such management interventions are warranted. As Murphy et al.^3^ noted, these may be among the most abundant aquatic snake species on the planet, occurring over vast areas. The key issue for this taxon is preservation of habitat (especially, mangroves) rather than specimen-based protection. Given significant economic benefits of snake-harvesting for local people, it is difficult to see value in reducing current levels of commercial trade.

## Data Availability

Data will be deposited in the Dryad Digital Repository upon manuscript acceptance. The datasets used and/or analyzed during the current study are also available from the corresponding author on request.
